# Unsupervised real-world knowledge extraction via disentangled variational autoencoders for photon diagnostics

**DOI:** 10.1038/s41598-022-25249-4

**Published:** 2022-12-01

**Authors:** Gregor Hartmann, Gesa Goetzke, Stefan Düsterer, Peter Feuer-Forson, Fabiano Lever, David Meier, Felix Möller, Luis Vera Ramirez, Markus Guehr, Kai Tiedtke, Jens Viefhaus, Markus Braune

**Affiliations:** 1 Helmholtz-Zentrum Berlin für Materialien und Energie GmbH, Albert-Einstein-Strasse 15, 12489 Berlin, Germany; 2 Deutsches Elektronen-Synchrotron (DESY), Notkestrasse 85, 22607 Hamburg, Germany; 3 Institut für Physik und Astronomie, University of Potsdam, Karl-Liebknecht-Strasse 24/25, 14476 Potsdam-Golm, Germany; 4 Intelligent Embedded Systems, University of Kassel, Wilhelmshöher Allee 73, 34121 Kassel, Germany

**Keywords:** Atomic and molecular interactions with photons, Characterization and analytical techniques, X-rays, Applied mathematics, Scientific data

## Abstract

We present real-world data processing on measured electron time-of-flight data via neural networks. Specifically, the use of disentangled variational autoencoders on data from a diagnostic instrument for online wavelength monitoring at the free electron laser FLASH in Hamburg. Without a-priori knowledge the network is able to find representations of single-shot FEL spectra, which have a low signal-to-noise ratio. This reveals, in a directly human-interpretable way, crucial information about the photon properties. The central photon energy and the intensity as well as very detector-specific features are identified. The network is also capable of data cleaning, i.e. denoising, as well as the removal of artefacts. In the reconstruction, this allows for identification of signatures with very low intensity which are hardly recognisable in the raw data. In this particular case, the network enhances the quality of the diagnostic analysis at FLASH. However, this unsupervised method also has the potential to improve the analysis of other similar types of spectroscopy data.

## Introduction

### SASE-FEL challenge

**Figure 1:**
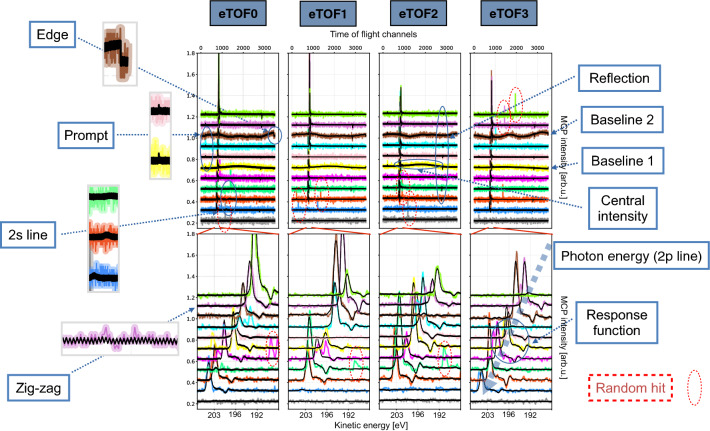
Eleven representative single-shot time-of-flight spectra (samples) obtained by the four OPIS electron spectrometers (eTOF 0–3): The grey traces at the bottom show no photo electron signal, whereas the other ten traces above contain Ne 2p photoelectron lines with successively longer time-of-flights, indicating decreasing FEL photon energy. The raw data is shown by coloured bold plots while the reconstruction of the corresponding samples is depicted by black thin lines. For better visibility the base lines are separated by a vertical offset of 0.1. The four upper panels show the full electron time-of-flight spectra, which is the input of the neural network, while a zoom-in of the corresponding region of interest, where the 2p line is expected, is presented in the lower four panels. The zoom-in axis is converted to kinetic energy of the photoelectron. Main features of the traces are labelled, such as peak position, random hits, baseline disturbances, a zig–zag structure, the prompt, 2s and 2p line, electronic reflection due to impedance mismatch on cable connection and the corresponding detector response function. These are reconstructed (apart from the random hits) and encoded in the latent space. The magnified insets represent features that are difficult to see in full scale. All scales are linear.

Free electron lasers (FEL) enable atomic and molecular science in the femtosecond to attosecond regime by creating highly intense photon pulses on that time scale. However, FELs which are based on the principle of self-amplified spontaneous emission (SASE)^1,2^, such as FLASH^3^, produce spatial, spectral and temporal pulse properties that are strongly fluctuating from pulse to pulse. Hence, a reliable photon diagnostic on a single-shot basis is essential for sound data analysis of scientific user experiments performed at such facilities. Post-experiment sorting of recorded data with respect to different properties, such as intensity or wavelength, can reveal signatures of physical processes otherwise obscured or even hidden in the data sets. A number of diagnostic instruments at FELs are used to measure the photoionisation of gas targets, such as the Gas Monitor Detector (GMD)^4,5^ for measurement of absolute pulse energy, THz-streaking^6,7^ for determination of the photon pulse time structure^8^, as well as the online photoionisation spectrometer OPIS^9,10^ (see Fig. 1) and the so-called cookie-box^8,11^ which use photoelectron spectroscopy to get information about the spectral distribution of the FEL radiation. These diagnostic methods have the advantage that they can be designed to be almost completely non-invasive. In a photoionisation process, due to the high FEL intensity, a significant space charge^10^ can be created in the ionised gas target in the interaction region of the instruments. This space charge even accumulates for high FEL pulse repetition rates, since the created target gas ions cannot dissipate fast enough by Coulomb repulsion or be replenished with fresh, unionised atoms before the next FEL pulse arrives. For instruments based on photoelectron spectroscopy, such as OPIS, space charge can distort the diagnostic measurement because it alters the kinetic energy distribution of the photoelectrons. To minimise such space charge-induced detrimental effects OPIS is operated at low target gas pressures. For this reason, OPIS’ single-shot spectra usually show low count rates and consequently photolines comprise only a small number of single-electron events, appearing as spikes in the spectrum, which are not clearly distinguishable from random noise spikes (see Fig. 1). In order to obtain meaningful wavelength results, a moving average scheme over variable time intervals is usually applied. Hence, reliable shot-to-shot information, which is important for experiments, could not be provided in the majority of cases in the past. We here present a method to reveal the photon properties in single-shot resolved mode, despite the low statistics, by employing artificial intelligence that takes advantage of a special type of autoencoder, which represents the data obtained by the diagnostics device in a compressed and comprehensible way.

### AI approach

Traditional analysis methods like principal component analysis (PCA) are robust and have proven their capability in various applications^10^ but can be limited by two main issues: (a) The method is linear und thus intrinsically unable to describe non-linear effects and (b) the representations of the data (the principal components and their scaling factors) are not necessarily easy to interpret. Scaling well with high dimensionality and being able to describe non-linear effects, neural networks became popular during the last decades as a powerful analysis tool in all categories of science^12^. Autoencoder (AE) networks^13^ built by layers of neurons are capable of compressing data to lower dimensionality, the so-called latent space. While a 1-layer AE network is equivalent to a PCA analysis^12^, problems of higher complexity and with non-linear effects can be handled by adding multiple layers of neurons to the encoder and decoder. When using such a network, the latent space representation cannot typically be easily used for knowledge extraction and has to be further processed in order to transform it into parameters that humans can interpret. This can be done, for instance, with another neural network. However, this process requires the setting of labels for training the network, i.e. attribution of the actual values of certain physical properties at the time of the measurement to the recorded data, which in our case as well as in many other applications are not available. Variational autoencoder^14,15^ networks (VAE) perform a sampling operation on a mean and standard deviation vector in the dimensional bottleneck of the network. By forcing these two vectors to be close to a normal distribution by the use of an additional term in the loss function, one creates a representation with a given value range and variation. By varying the latent space within these limits it is possible, with the decoder part of the network, to create artificial data samples which represent possible measurement results. This idea was implemented by so-called $\beta$-VAE-networks^16^ in which the disentanglement-term in the loss function is scaled by a factor, called $\beta$. Thus, it is possible to balance the weight between a perfect reconstruction (i.e. mean-square-error deviation of the raw and the reconstructed data) and perfect disentanglement of the latent space vector components, creating a compromise between the disentanglement ($L_{\text {dis}}$) and reconstruction quality ($L_{\text {rec}}$), both represented in the overall loss function ($L_{\text {all}}$):

1 $$\begin{aligned} L_{\text {all}}=L_{\text {rec}}+\beta \cdot L_{\text {dis}} \end{aligned}$$

Generally, finding the best absolute value of $\beta$ is challenging^16,17^. $\beta$ strongly depends on the data, i.e. on the noise level, the size and shape of the region of interest, and on what measure is used to evaluate the reconstruction quality.

## Experiment

### FLASH and OPIS

FLASH^3^ operates in a so-called burst mode pattern, generating bunch trains with a burst repetition rate of 10 Hz. Each bunch train consists of up to several hundred single photon pulses, depending on the bunch repetition rate of up to 1 MHz. At FLASH2^18^, pulse energy and pulse duration range over 1–1000 µJ and 10–200 fs, respectively, covering a wavelength range of 4–90 nm. For online FEL wavelength monitoring with OPIS (see Fig. 1, for details see^9^) a noble gas target, in our study neon (gas pressure $4.4 \times 10^{-7}$ mbar), introduced into the interaction chamber is ionised by the FLASH pulses. The kinetic energy $E_{kin}$ of the generated photoelectrons is measured by four independently working electron time-of-flight spectrometers (eTOF). With the knowledge of the binding energy $E_{bin}$ of the excited orbitals, for our study neon 2p and 2s, one can calculate the photon energy $E_{pho}$ via

2 $$\begin{aligned} E_{pho}=E_{kin}+E_{bin} \end{aligned}$$

In the eTOF, the photoelectrons travel along a drift tube of 309 mm in length and are then detected by microchannel plate (MCP) detectors. Retarding voltages can be applied to the drift tubes in order to decelerate the photoelectrons and therefore increase the energy resolution of the eTOF spectrometers.

### Data

Time traces of the amplified signals from the MCP detectors are recorded by means of fast analog-to-digital converters (ADCs) with a sampling rate of 7 GS/s and 8-bit vertical resolution. Each single-shot spectrum consists of 3500 ADC channels and the aggregate of the four eTOF spectra represents one training data sample with a dimensionality of $4 \times 3.5\,\text {k = 14 k}$ (including only an estimated number of electrons ranging from 0 to 20). Some examples are presented in Fig. 1. The intensity of the photoelectron lines in the recorded TOF spectra are comparable in all four eTOFs, being on average within 15 % of the amplitude (standard deviation). However, in single-shot spectra the photoline intensities vary significantly between the four eTOFs due to statistical effects. Figure 1 depicts a series of normalised single-shot data corresponding to different values of the photon energy of the FEL radiation, for varying time of flight of the neon 2p electrons. A time frame of continuous wavelength monitoring is chosen in which the OPIS operation parameters (gas target, chamber pressure, spectrometer retardation) remained unchanged. In this time interval, the FEL photon energy was scanned between 214 and 226 eV with a given irregular pattern. In OPIS, neon was used as a target gas and the retarding voltage was set to 170 V, resulting in a final reduced kinetic energy of 22.4 to 34.4 eV and 0.0 to 7.5 eV of the detected 2p and 2s photoelectrons, respectively. Roughly 40 million samples were recorded.

### Neural network

The ultimate goal is to train a network that delivers all desired information in a low dimensional latent space, i.e. each latent space component should represent a property of the underlying core principle that can be interpreted by the human mind and therefore can be directly used as information for the experiments. For the loss function, mean-squared-error (MSE) is used as a criterion for reconstruction quality. The disentanglement is described by the Kullback–Leibler (KL) divergence^20^ of the mean and standard deviation vector compared to a normal distribution. In order to automatically stay within the value range of [0,1] the output layer is activated with a sigmoid function. In order to optimise the hyperparameters of the neural network about 700 different networks were trained. The best performance was achieved with fully connected and Mish-activated^22^ layers with the decoder and encoder consisting of 5 and 4 layers, respectively. Batch sizes of 252 were used in combination with the Adam optimiser^23^ and a scheduled decreasing learning rate ranging from $10^{-5}$ to $10^{-7}$ throughout 25 k epochs. The optimised value of $\beta$ is 0.034. Of the 40 million data samples recorded in total, 33 million were used for training, 1 million for validation and the remaining 6 million represent the test data utilised outside of the training process. The best performance of the encoder and decoder is achieved when the layers are chosen such that the dimensionality is reduced with the same factor for each layer, which means for 5 layers and a 12-dimensional latent space, called *z*, the dimensionalities of the layers are

3 $$\begin{aligned} 14000\rightarrow 2824\rightarrow 579\rightarrow 117\rightarrow 24\rightarrow 12 \end{aligned}$$

The step from 24 to 12 is the sampling operation. The decoder is the mirrored version of the encoder excluding the sampling operation. The number 12 was derived by training a network starting with only a one-dimensional *z* and then successively increasing the size of the dimensional bottleneck. For a size larger than 12, the final loss value failed to significantly improve. We will use the notation $z=\left\{ z_{0},z_{1},z_{2},...,z_{11}\right\}$ to address the individual components $z_{i}$ of the latent space.

## Results

### Creating labels

The aim of OPIS measurements is to reveal values of certain physical quantities. To analyse whether the network found a latent space representing those quantities, labels are created by conventional analysis performed on the raw data. To provide reliable labels the data has to meet specific criteria, which is only applicable for a small fraction of the available data. For example, for the flight time of the photoelectrons, i.e. the photoline position on the TOF-scale (referred to with the label $T_{0,1,2,3}$), a conventional least-square line profile fit analysis of the strongest peak in each of the four eTOFs has been performed. Here, the criteria for discrimination of a valid photoelectron line feature from noise or random electron hits was defined such that (a) the peak amplitude has to be larger than a threshold for minimum intensity (0.5 on the scale in Fig. 1) and (b) the peak centre positions have to lie within a small TOF range (15 TOF-channels). Applying this filter reduces the size of the test data dramatically, but returns high quality data. Roughly 3 % of the data fulfills this criteria and can contribute to the comparison between the labels and the latent space. The labels for the individual intensities for each eTOF called $I_{0,1,2,3}$ are additionally created in the process of the peak fit procedure. The beam position of the FEL in the plane perpendicular to the propagation axis is also fluctuating. In order to have a robust and simple label for these pointing variations, the 2p electron time-of-flight difference is calculated, resulting in $P_{02}$ (eTOF0 compared with the opposite positioned eTOF2) and $P_{13}$ (eTOF1 compared with the opposite positioned eTOF3). This is explained in detail in the supplementary information (SI). The “Baseline 1” disturbance $B_{1}$ can be identified by evaluating eTOF0 regarding discontinuities, i.e. the sharp ” edge” feature at high time-of-flight values. It is identified by calculating the sum of the intensities of 40 ADC-channels before the edge divided by 40 after the step in the trace baseline. The second disturbance $B_{2}$ (see “Baseline 2” and “Central intensity” in Fig. 1) is a broader feature covering the central part of each TOF-spectrum. It is identified and labelled by summing up the central part of the spectra which is then divided by the mean of the data in spectral regions at the beginning and the end of the spectrum. For the data acquisition the effective sampling rate of 7 GSamples/s is achieved by time-interleaving four ADC chips, sampling with 1.75 GS/s each. We realised that the network was encoding a correlation directly pointing to systematic interleaving deficiencies: In large parts of the data, the gain of the respective interleaved ADCs of each eTOF channel is not identical which creates a characteristic zig–zag-structure in the data (see zoom-in and “zig–zag” in Fig. 1). This can be easily labelled by adding all odd and all even ADC channels separately and then dividing these two sums, resulting in the labels $L_{0,1,2,3}$. For the photon energy, one OPIS-independent value is the set wavelength parameter $\lambda _{FEL}$ which only represents the nominal wavelength corresponding to the FLASH accelerator and undulator setup. The real FEL wavelength can have a certain offset, mainly due to two factors: Firstly, the electron beam energy in the undulator section may deviate from the energy value measured in the accelerator section due to beam steering components such as the FLASH2 extraction and bunch compression chicanes^18^. Secondly, the electron beam orbit can deviate from the nominal orbit in the undulator section, especially if the variable gap undulators are tuned for wavelength scans. Furthermore, the wavelength fluctuates due to the SASE process within a bandwidth of typically $\sim$ 1%^3^, which in our case corresponds to a photon energy bandwidth of about 2 eV. Therefore, the label $\lambda _{FEL}$ is an ‘estimated’ label with only moderate significance for the single-shot photon energy. Additionally, a magnetic bottle experiment^21^ was performed in parallel with our study and its data is used as a cross reference for the wavelength, which is presented in the SI.

### Reconstruction and interpretation of the latent space

**Figure 2:**
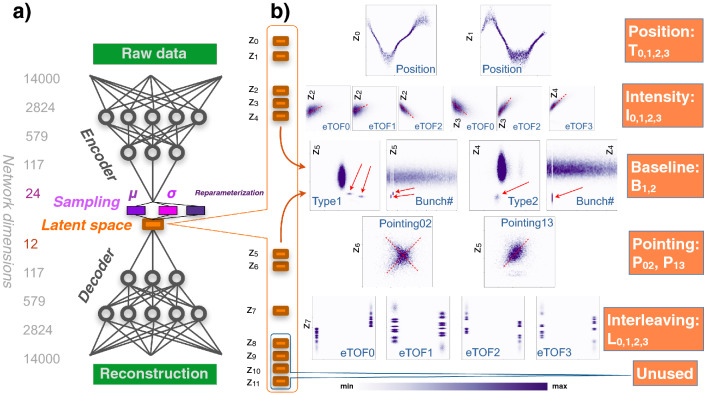
The structure of the $\beta$-VAE-network (**a**) and the unsupervised encoding of the underlying core principle (**b**), i.e. position, intensity, baseline, pointing and interleaving, is shown. The density plots represent the dependency of the latent space vs. the labels, which were derived by traditional data analysis using the high quality data (3% of the data set). The values in the corresponding axis ($z_{i}$ and labels) are min–max-normalised for the processed test samples. All scales are linear.

These labels, that result from the aforementioned feature engineering process, are compared with the $z_{i}$ values that the network derives for the data in Fig. 2. The reconstruction quality (black curves in Fig. 1) is impressively high for a 12-dimensional bottleneck. The network finds the correct position of the 2p photoelectrons, it reconstructs the individual MCP-response function for each of the 4 eTOFs, it discards random uncorrelated events and is also able to reproduce the baseline disturbance. In addition to these findings, the neon 2s line is contained in the reconstruction only in cases when the photon energy is in fact sufficiently high enough to overcome the used retarding voltage of the flight tubes. Given that for our data the ionisation cross section is $\sim$ 5 times lower for Ne 2s compared to Ne 2p in the photon energy range of 214 eV to 226 eV and that the 2s photoline intensity spreads over a larger TOF interval, this is an impressive result^19^. Ne 2s signatures can hardly be identified in the raw data by eye or using conventional analysis methods. Equally impressive is the reconstruction of the so-called prompt signal, which is created by scattered photons hitting the MCPs and hence produces another tiny peak feature at a fixed TOF-position. This signal marks the reference t = 0 for the determination of the photoelectron flight time and is therefore of high importance.

A critical piece of information for most experiments at SASE-FEL sources is the single-shot central photon energy. In OPIS measurements it corresponds to the peak position in the eTOF-spectra which is encoded in two components of *z*, namely $z_{0}$ and $z_{1}$. In the $z_{0,1}$-position maps it exhibits a dependency resembling sine and cosine functions, respectively. However, the position is not encoded in a perfect sine-cosine or circle manner. This is combined to a phase $\phi$ defined by:

4 $$\begin{aligned} \phi =\arctan \frac{z_{0}}{z_{1}} \end{aligned}$$

**Figure 3:**
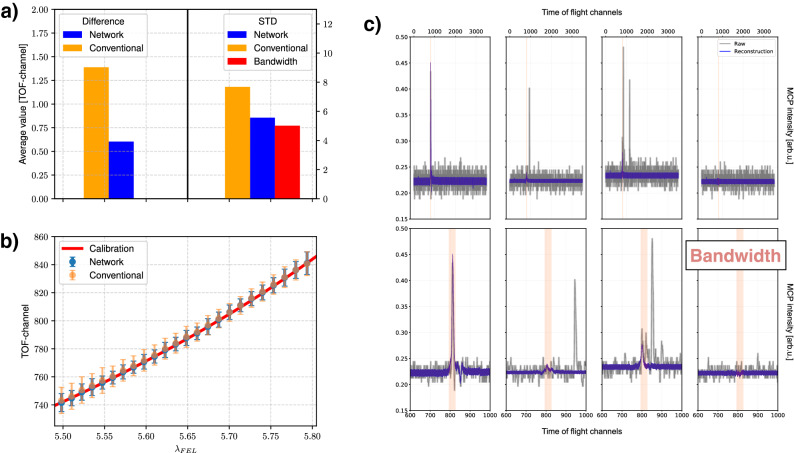
Comparison of the performance between the traditional analysis and the neural network: (**a**) The average difference of the network’s prediction of the time-of-flight position (blue) compared with the expected position at the given $\lambda _{FEL}$ from the calibration is significantly lower than the average error given by the traditional analysis (orange). The expected bandwidth is transformed to the STD in TOF channels (red). The STD from the neural network’s predictions are almost identical to the bandwidth. (**b**) For the 25 $\lambda _{FEL}$, the determined TOF-positions of the network and of the traditional analysis are compared with a calibration curve according to the OPIS instrument calibration, which was independently determined in the instrument commissioning campaigns. (**c**) An example shot (grey) is shown which has several peaks at different positions in the eTOFs. The expected position within the given bandwidth is shown in red. While traditional analysis cannot decide which of the peaks to designate as real photoelectron signal, the network reconstructs the peaks at the correct position while ignoring all other peaks in the raw data.

In order to provide the most accurate wavelength, $\phi$ is corrected with an additional neural network. A fraction of the data (3% high quality data) where all four eTOFs provide the same information for the wavelength, i.e. clear photolines at similar positions, is used to train a fully-connected multi-layer-perceptron (MLP)^24^. This MLP projects $\phi$ to the average least-square fitted TOF-positions of the 2p peaks from all four eTOFs (see Supplementary Information). The performance of the method is evaluated threefold: It is compared to (a) the results of the conventional data analysis (see “Methods” section), (b) $\lambda _{FEL}$ and (c) the center of mass of the magnetic bottle experiment (see Supplementary Information). The comparison with $\lambda _{FEL}$ is made by using OPIS’ calibration curve which is shown in Fig. 3b for the network and the traditional analysis. The results are summed up in Fig. 3a. The average difference of the network’s prediction in TOF-channels is smaller by a factor of 2 when compared to the conventional method. The estimated bandwidth of the FEL is translated to a standard deviation value (STD) in TOF-channels. This STD of the bandwidth is close to the STD of the network’s predictions, whereas the conventional result differs more significantly. To showcase how the network outperforms the conventional analysis, Fig. 3c depicts a shot which is difficult to analyse. Multiple peaks with similar amplitude are appearing at different TOF positions. $\lambda _{FEL}$ including the bandwidth is shown to indicate the region where the photoelectrons are expected. The network reconstructs the peak in the correct region, presented in Fig. 3c. Contrastingly the traditional method struggles to identify the correct peak(s). As an SASE-fluctuation independent comparison, the predicted wavelength is also compared to the center of mass of the 2p photoline of sulfur from 2-Thiouracil in the magnetic bottle experiment, which was running in parallel to our study. Here, a good agreement is also found and this is presented in the SI.

Besides the wavelength retrieval, multiple other features are encoded in the latent space during the unsupervised training process. The network encodes the intensity distribution of the 4 eTOFs in $z_{2}$, $z_{3}$ and $z_{4},$ which is plotted in Fig. 2. $B_{1}$ and $B_{2}$ are encoded in two separate components of *z*, namely $z_{4}$ and $z_{5}$, as shown in Fig. 2. Interestingly, $B_{1}$ only occurs in two specific bunches of the pulse train and $B_{2}$ is even limited to only one bunch (see Bunch-No. vs $z_{i}$ maps), indicating synchronised electronic noise induced from the accelerator environment as a cause. $B_{1}$ and $B_{2}$ are encoded in an on/off-state and therefore $z_{4}$ and $z_{5}$ can use an extreme value region for “on” and while the baseline disturbance is “off” they can use the rest of the value range for the encoding of a different feature. As a result, $z_{4}$ also encodes the intensity of eTOF3 while $z_{5}$ is also encoding $P_{13}$. The network uses the sixth dimension of *z* for the other pointing related label $P_{02}$. The linear dependency of $z_{5}$ vs. $P_{13}$, combined with the cross-like dependency of $z_{4}$ vs. $P_{13}$, can now be used to determine the variation of the spatial beam position which can also be an important parameter for the experiments. $L_{0,1,2,3}$ are fully encoded in $z_{7}$. The components $z_{8-11}$ are only influencing the reconstruction in a tiny way and therefore are considered unused. However, reducing the dimensionality of the latent space increases the overall loss resulting in a more complicated encoding of the handcrafted labels.

**Figure 4:**
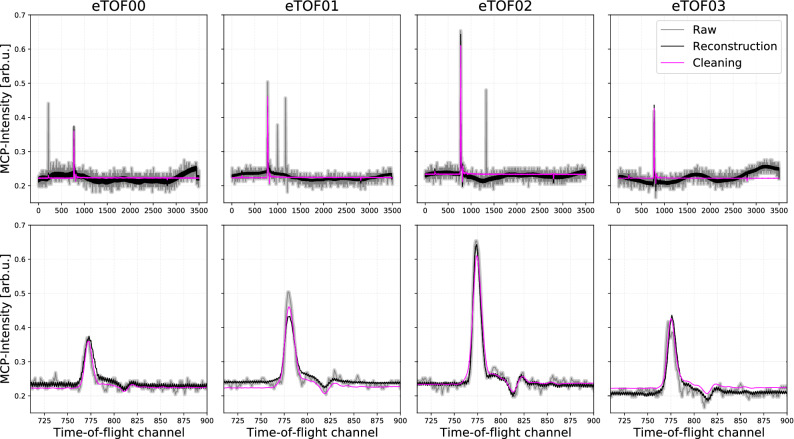
Data cleaning example: The raw data (grey) is reconstructed via the network as shown in black. All random hits are discarded, the noise level is reduced and the prompt signal is reconstructed. A modification of the latent space allows for eliminating the interleaving issue and a removal of the baseline disturbance (magenta).

### Data cleaning

The reconstruction of the data by the network alone, already automatically removes all the random hits from the raw data. Additionally, the noise level of the baseline is strongly reduced. Finally, with both parts of the network, the encoder can be used to get the 12D-representation of the individual samples and consequently one can selectively clean the compromised data of all these effects, which is shown in Fig. 4. Since the latent space representation is understood, one can just change $z_{7}$ from 0.8 (which was determined by the network in order to achieve the best reconstruction of this specific sample) to the average value of 0.0 and then by running the decoder with this modified $z_{7}$ value it is possible to eliminate the interleaving effect. A similar procedure (see Supplementary Information) can be used to remove the baseline disturbance.

### Outlook

In order to fully exploit the high-repetition rates of FEL machines with superconducting accelerators, which deliver FEL radiation with highly fluctuating photon properties due to the SASE operation mode, information about the essential parameters are needed on a single-shot basis. Ideally, this information should be provided by entirely independent diagnostic devices, which can be operated in parallel to the running user experiment. This way, the best possible analysis—even in a near real-time fashion—can be enabled, allowing for all possibilities of data sorting, binning and similar methods, in order to reveal the dependencies on the photon properties for the physical process under investigation. This is especially important for photon-hungry experimental techniques, such as coincidence measurements, which rely on the accumulation of a large number of single photon interaction events. Blurring or even disguising dependency effects by averaging data samples covering a spread of different values of photon properties can be avoided. OPIS in combination with the trained $\beta$-VAE network can provide such ability and thus enables the use of the FEL property “wavelength” as an independent sorting parameter for any experimental data analysis. The next steps will be to train more general networks. The OPIS operation parameters, i.e. the target gas species, the chamber pressure and the retarding voltages on the eTOFs, have been kept at fixed values for the results that are presented in this work. We have recorded and will record spectra for a variety of combinations of these parameters. First, dedicated networks will be trained for different operation parameters. In this case, for each operation mode a specific network can be used for online analysis. Second, only a single network will be trained for all operation parameters, allowing the use of the same network for all operation modes. These two approaches will then be compared.

## Conclusion

We have shown that an optimised $\beta$-VAE-network is capable of finding the underlying core principle of high-dimensional photoelectron time-of-flight spectroscopy data without any a-priori knowledge in an unsupervised way. All raw data with low signal-to-noise ratio is denoised and random hits not correlated to the observed photoionisation processes are discarded. As a consequence, the reconstructed spectra are of a much higher quality and in certain cases can very clearly show photoelectron features which are obscured in the raw data and cannot easily be processed by conventional analysis methods. The representation in the latent space covers all the main intrinsic physical properties of the spectrum, providing direct access to essential information such as the single-shot FEL wavelength. The inference time of the trained network is fast and therefore it can be deployed as an online tool during photon diagnostics measurement, providing crucial information for FLASH user experiments in real-time. This will enable or improve the on-the-fly data analysis which helps to enhance the efficiency of a beamtime. For instance, by monitoring the data quality in terms of statistics, for the effect under investigation, one can optimise the recording time and evaluation of the findings. This concomitant analysis affords the user the ability to adapt the measurements on the fly throughout the experimental campaign. Furthermore, any offline post-experiment data analysis will also benefit from the labels provided by the $\beta$-VAE-network. In this respect, the ability to isolate or eliminate certain properties of the data by setting the values of the VAEs representing those properties to zero may be very useful for a detailed in-depth analysis of the data set.

## Methods

### OPIS time-of-flight calibration

For accurate wavelength measurements with OPIS an instrument calibration is required. In OPIS commissioning campaigns conversion functions which assign kinetic energy to measured time-of-flight values have been empirically determined for each retardation voltage setting. In these calibration measurements either the photon energy or the electron kinetic energy was precisely known (Eq. (2)). This has been achieved by simultaneous measurements, together with an optical grating spectrometer as a reference, as well as using the intrinsic calibration capabilities by means of Auger processes. Auger electrons are emitted with a fixed kinetic energy corresponding to the difference of the two electron orbitals involved in the Auger transition and hence can serve as direct kinetic energy markers in the TOF spectrum. Furthermore, schemes can be used in which the FEL wavelength is tuned until the TOF position of a photoline of a particular orbital precisely matches an Auger line position. This also determines the wavelength and therefore defines the kinetic energy at the TOF position for other photoelectron lines in the same spectrum. More detailed information about the OPIS calibration can be found in Refs.^9,10^.

### Hyperparameter optimisation

Table 1 shows the hyperparameter space that was explored while $\sim$ 700 networks were trained. The batch size, the $\beta$ parameter, the learning rate and the samples per epoch were tested at a fixed value as well as within a scheduling process. Apart from assessing the overall loss, which is a combination of the MSE-reconstruction loss and the KL-divergence disentanglement loss of the latent space, the evaluation of the network, with regards to the interpretability of the latent space with the handcrafted labels, was performed via least-square fitting as shown in Fig. 2. For the reconstruction loss, absolute error (AE) and binary cross entropy (BCE) were also tested. The components of *z* in Fig. 2 (and the text) are reordered for better readability. In the case of the stochastic gradient descent optimiser (SGD), the momentum was tested from 0 to 0.9. The 40 million samples are divided and randomly shuffled into 40 single hdf5-files each containing one million samples. 33 of these files are used for training, one million samples as validation data during the training process, and the remaining six million to test the trained network afterwards. For data loading purposes, one epoch is defined as an optimisation step within which the network processes one file, i.e. one million samples. During training, the network continues training with the same one million samples for a fixed number of epochs until the data is replaced by another one million samples from another file and so on. Memorisation of the data, with regards to a fixed one million sample portion of the data, is only observed in very deep networks and also only after a couple of thousands epochs. Due to this effect, the training data in memory is replaced every 10 epochs, ensuring that overfitting does not occur, whilst still allowing for fast data transfer to the GPU which is used to train the network. An additional indication that this way of training is not compromising the final result is that no abrupt changes are observable in the loss function if the data set is replaced after 10 epochs. If the number of epochs for the same data is set to 1, the process can be interpreted as processing the entire training data of 33 million samples each 33 epochs. The data was min-max-normalised, i.e. the 8-bit vertical integer range of [0,255] was transformed to float values in the interval [0,1].

**Table 1:** Hyperparameter optimisation: in order to find the best network, the shown hyperparameters were varied in the given region.

Hyperparameter	Tested	Chosen
Batch size	8–32 k	256
Optimiser	Adam, SGD	Adam
Learning rate	$10^{-2}$–$10^{-8}$	Scheduled: $10^{-5}$ to $10^{-7}$
Activation function	ReLU, sigmoid, tanh, Mish	Mish
Output activation	None, sigmoid, ReLU, Mish	sigmoid
Samples per epoch	10 k–33 M	1 M
Epochs on same samples	1–10,000	10
Training data size	1–33 M	33 M
$\beta$ parameter	0–10	0.034
Layers encoder	1–10	5
Layers decoder	1–10	4
Max. neurons per layer	20 k	14 k
Latent space dimension	1–20	12
Normalisation	None, Min-Max, Std	Min–Max
Reconstruction loss	BCE, MSE, AE	MSE

### Phase correction by the MLP

The MLP for the phase correction of $z_{0}$ and $z_{1}$ has the following network architecture

5 $$\begin{aligned} 1\rightarrow 100\rightarrow 60\rightarrow 36\rightarrow 21\rightarrow 12\rightarrow 1 \end{aligned}$$

while the input is the phase and the prediction target is given by the average TOF-position derived by fitting all 4 eTOF spectra. It was trained over 2000 epochs with 200 k samples, a batch size of 100 and a learning rate of $10^{-5}$, while Mish-activation and the Adam-optimiser were used. The data was not normalised. The prediction quality was measured in MSE.

### Conventional data analysis

Multiple methods were tested for processing the single-shot raw data in a robust and efficient manner. The comparison was made regarding how well the the data agreed with $\lambda _{FEL}$. The best results were achieved by an iterative procedure which only analyses the region of interest, TOF-channels [600, 1000], corresponding to the zoom-in region in Fig. 3c. First, a threshold of 0.2 (with respect to the values shown in Fig. 1) is set to determine all possible peak positions in all four eTOFs (multiple peaks in one eTOF are possible). These peak positions are integer values of the maximum position(s). Second, the peak positions of all detectors are compared. If there is more than one peak in the same window of 20 TOF-channels for multiple detectors, further processing of these peaks is performed. Otherwise, if the amplitude of one peak is higher (by an absolute value of 0.15) then further processing is only performed on this single peak. If not, then processing continues on all found peaks. All remaining peak positions are then optimised by calculating the center-of-mass of the peak (with floating point precision). Additionally, it was also checked to see whether other analysis methods, e.g. least-square optimisation fit routines, could be more suitable. It turns out that there is no advantage when using these other methods, but they create the disadvantage of a large increase in computing time. The average value of all determined peak positions is then taken as a final result.

## Supplementary Information

Supplementary Information.

## Data Availability

The datasets used and analysed during the current study as well as the code for the training process of the neural network are available from the corresponding author on reasonable request.
