# Supplementary material for: Unsupervised real-world knowledge extraction via disentangled variational autoencoders for photon diagnostics
Source: Sci Rep. 2022 Dec 1;12:20783. doi: 10.1038/s41598-022-25249-4 (PMC9715554; doi:10.1038/s41598-022-25249-4)
Supplement: Supplementary file 1 — Supplementary Information. [file 41598_2022_25249_MOESM1_ESM.pdf]

Supplementary Information (SI): Unsupervised real-world knowledge extraction via disentangled variational autoencoders for photon diagnostics

Gregor Hartmann, Gesa Goetzke, Stefan Düsterer, Peter Feuer-Forson, Fabiano Lever, David Meier, Felix Möller, Luis Vera Ramírez, Markus Guehr, Kai Tiedtke, Jens Viefhaus and Markus Braune

Disentanglement without a-priori knowledge

In this section, we describe how disentanglement is created and evaluated. It is important to keep in mind that the encoding (Fig. 2) and the reconstruction (Fig. 1) are achieved without any supervision. The network is supplied only the raw data with no further information, what we call "without a-priori knowledge". The dimensionality of the raw input data (14k) is compressed by the encoder in Fig. 2 to a dimensionality of 12 in the latent space. In the previous layer (labeled Sampling in Fig. 2) the network maps the data to a dimensionality of 24, representing the location (μ) and the scale (σ) of a normal distribution. This distribution is then compared to a normal distribution at location 0 and with a scale of 1 via the KL-divergence which is scaled by the β-parameter in the disentanglement loss. In order to visualise this effect, Fig. S1 shows the dependency maps of the latent space for a selected 3x3 section of the full 12x12 map for different values of β. By achieving a Gaussian distribution in these maps, one creates a situation where features that vary independently in the input should also vary independently in the latent space. If this is the case, then these features are considered disentangled in the compressed latent space. Of course, these maps cannot be used as the only criteria for evaluation. For large β values, the entire network just maps all inputs to perfect Gaussian distributions, whilst only reconstructing the average spectrum. Therefore, the other part of the overall loss function, the reconstruction loss, is necessary. From the latent space, the decoder reconstructs the input (the raw data) and an MSE-reconstruction loss can be calculated. No random effects, like noise or random hits, fit through the dimensional bottleneck and are therefore ignored in the reconstruction.

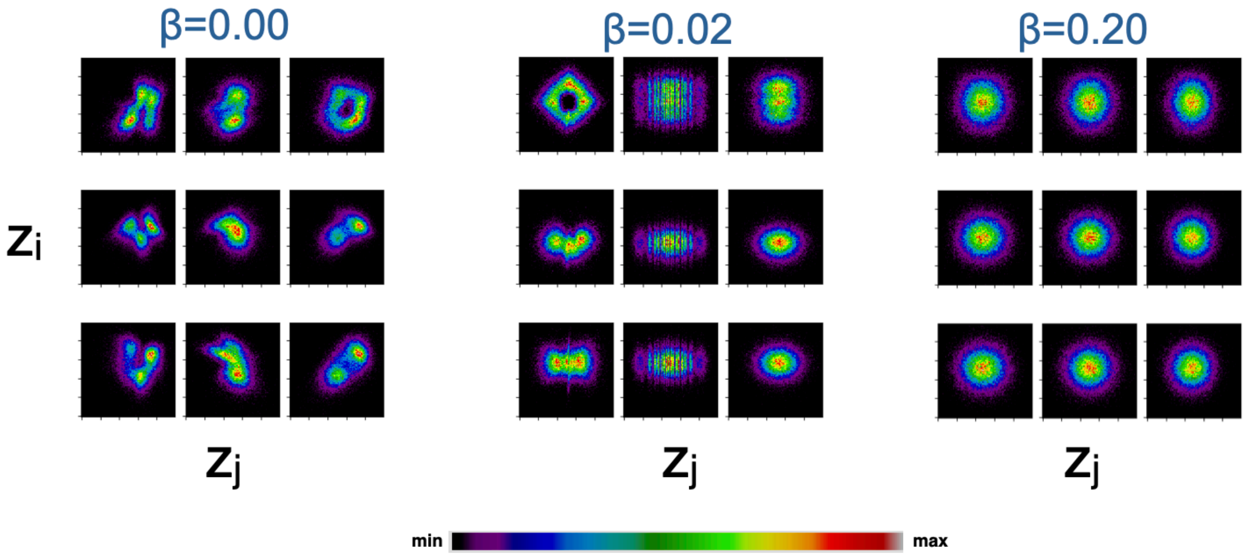

Figure S1: Dependency map showing 3x3 components of z for different values of beta. While complicated dependencies arise for a vanishing β, higher values create gaussian-like 2D-distributions. The disentanglement loss is evaluated in the shown dependencies (for all 12 components of z).

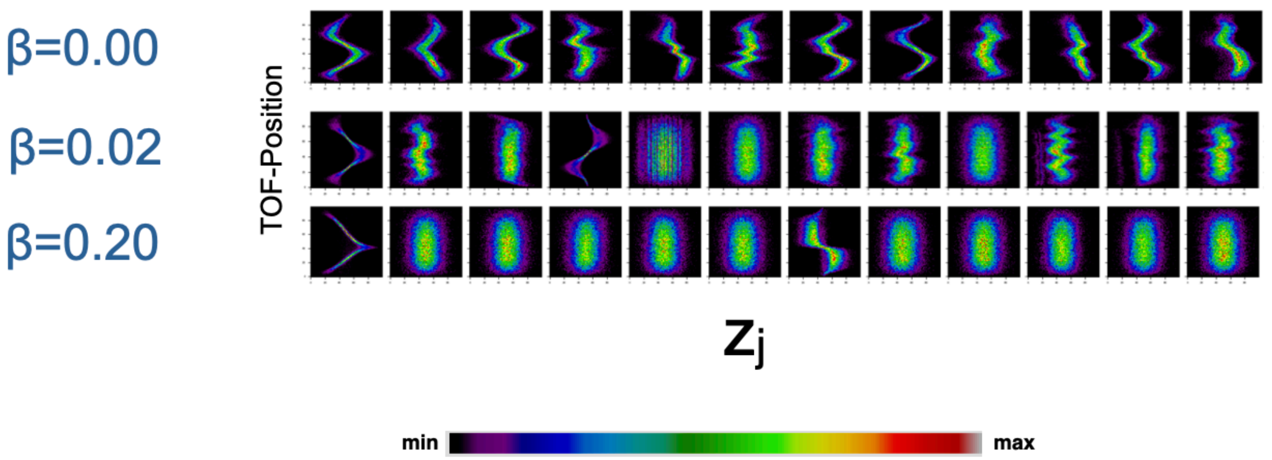

Figure S2: Encoding of the TOF-position vs. the latent space components for multiple values of β.

In addition to evaluating based purely on the loss, the compressed state is also evaluated via a comparison with the handcrafted labels, described in the main text and shown in Fig. 2. For the TOF-position, the influence of β is shown in Fig. S2. Ultimately, a value of 0.034 was chosen for β (and is used for all the plots in the main article). This value creates a situation where the 'position' is encoded in just two components of z and other key features of interest are encoded in such a way that they are easily accessible (see Fig. 2 of the manuscript).

Pointing

Fig. S3 shows the geometry of the four eTOFs of OPIS as well as the definition of the beam pointing parameters

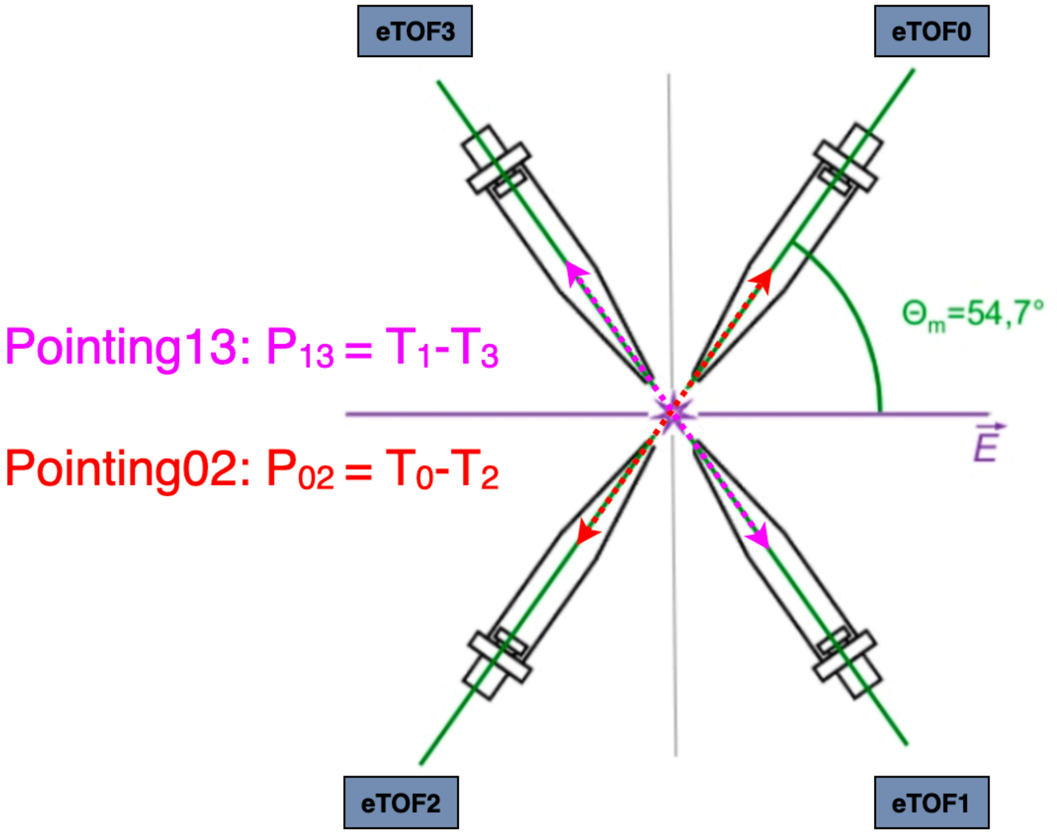

Figure S3: OPIS' geometry and the definition of the pointing parameters.

Data cleaning

The component z_4 encodes both I_3 (intensity) and B_2 (baseline). In order to remove the baseline disturbance via latent space manipulation and still maintain a high reconstruction quality, one has to follow the procedure shown in Fig. S4. If the disturbance is on, I_3 is encoded in the interval [0,0.25] as a linear dependency. The disturbance can be removed, without changing the peak intensity, by adjusting the z_4 value to match the corresponding value on the linear intensity dependence in the interval [0.4,1]. This is visualised by the vertical arrow in Fig. S4.

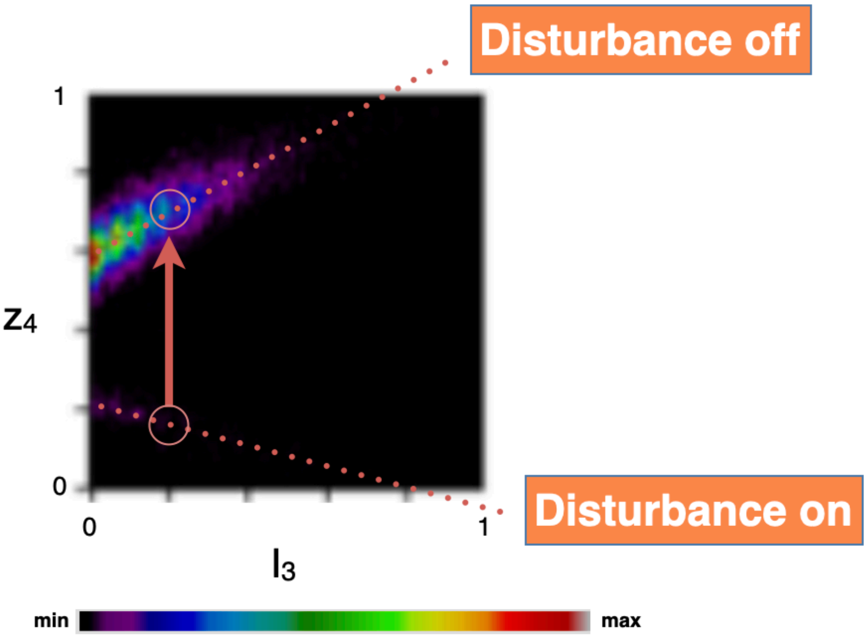

Figure S4: Procedure for baseline disturbance removal.

Phase correction

The information regarding the photoline position in the time-of-flight spectra is encoded in z_0 and z_1, which show a circle-like dependency as plotted in Fig. S5a, while ϕ along that circle is the position of the 2p photoelectrons. Fig. S5b shows the use of only high quality data, in which all 4 eTOFs contain the same information for the wavelength. Here, ϕ is compared with the least-square-fit position. ϕ is corrected by an additional neural network to compensate for the wiggles presented in Fig. S5b as described in the method section.

Magnetic bottle comparison

Although the data of the magnetic bottle also has low statistics (see Fig. S6), it is affected by SASE-fluctuations in the same way as OPIS. In the magnetic bottle experiment, the center of mass of the 2p photoline of sulfur from 2-Thiouracil is calculated in TOF-channels and compared with the predicted wavelength (WL) from the OPIS experiment. This was evaluated using multiple measures and shows a good agreement. The dependency curve (plotted in red in Fig. S7) is a) assumed to be linear in an easy and robust way or b) fitted with a polynomial approach. The agreement of the fit function vs. the predicted WL is evaluated in absolute and quadratic distance. In all defined errors (fit functions and distance definitions), the agreement of the predicted WL is higher than the agreement with the set wavelength λ_{FEL}.

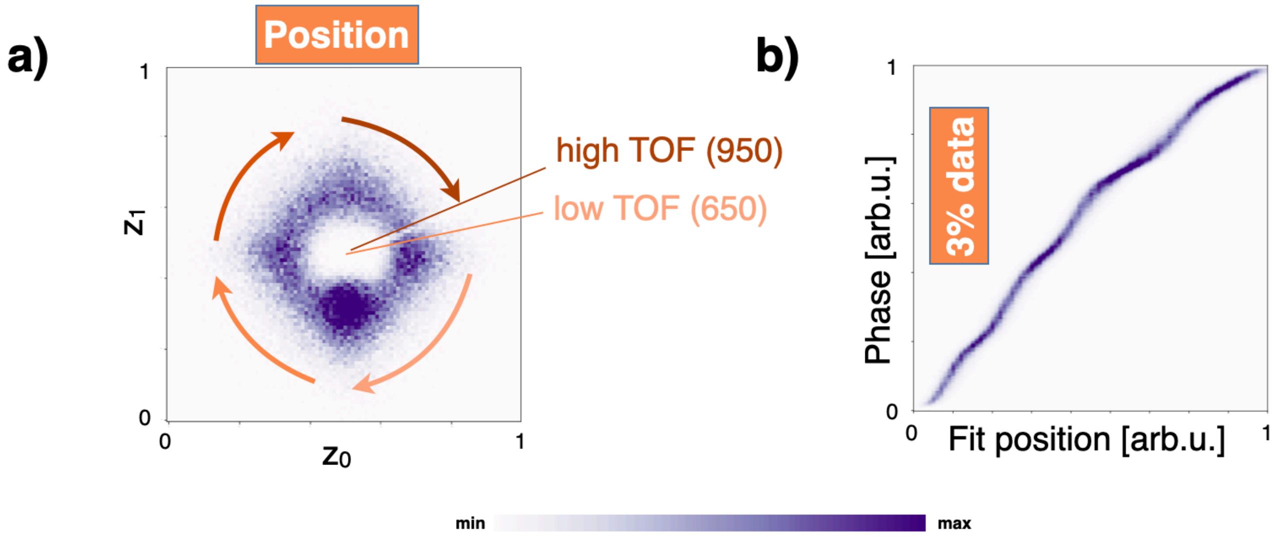

Figure S5: a) Position encoding along a circle-like shape. b) The wiggles caused by non-circle elements are shown in phase vs. fit-position dependency. An MLP is trained to map this dependency on a linear dependency.

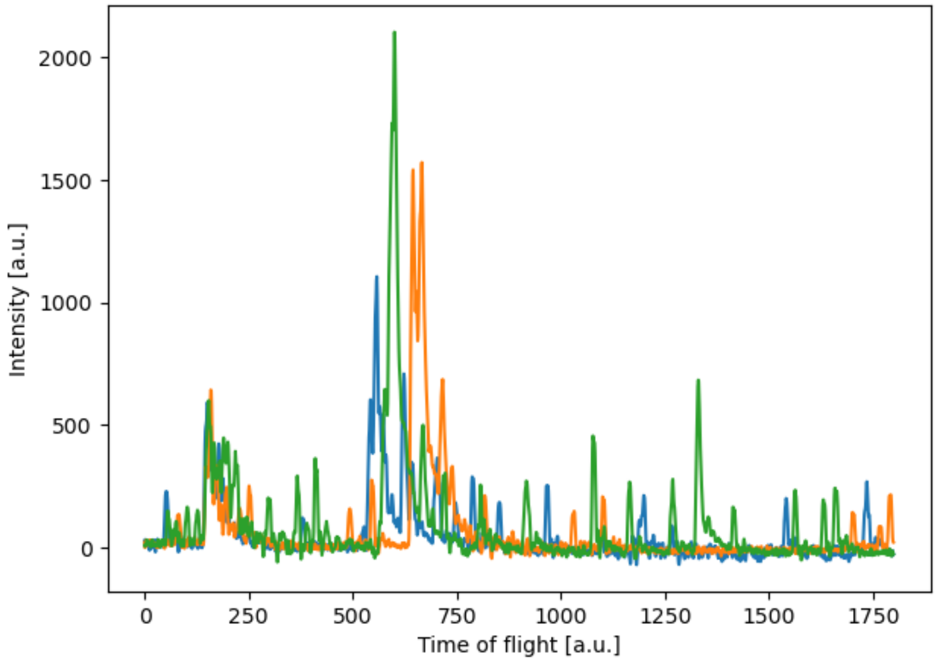

Figure S6: Three single-shot spectra from the magnetic bottle experiment. While valence shell electrons have a time-of-flight of ~200, the electrons used for photon energy determination arrive in the region [500,700].

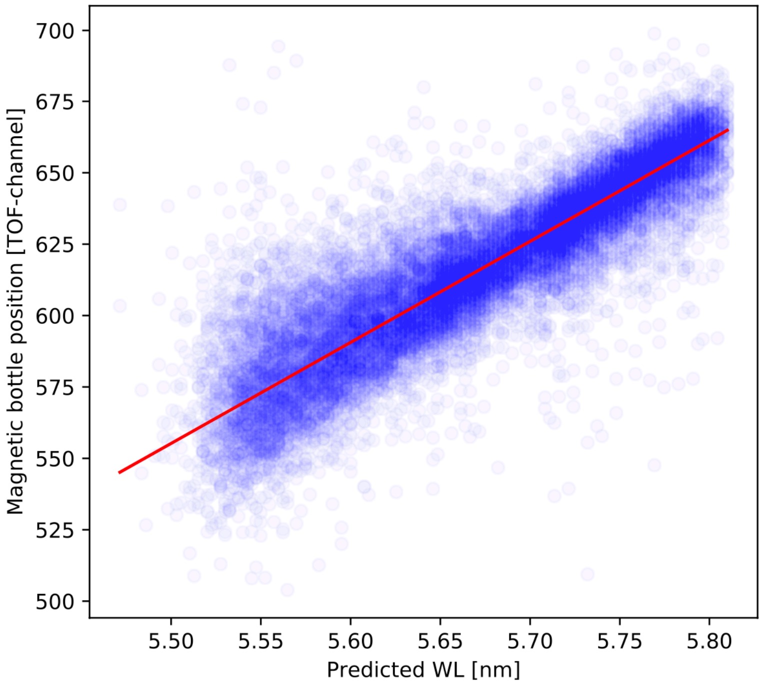

Figure S7: Comparison of the predicted wavelength from the neural network with the center of mass of the magnetic bottle (blue dots). In this example the dependency is assumed to be linear (red line).
